# Incorporation of the anteroposterior lumbar radiographs in the modified Stoke Ankylosing Spondylitis Spine Score improves detection of radiographic spinal progression in axial spondyloarthritis

**DOI:** 10.1186/s13075-019-1913-z

**Published:** 2019-05-24

**Authors:** Maria Llop, Valeria Rios Rodriguez, Imke Redeker, Joachim Sieper, Hildrun Haibel, Martin Rudwaleit, Denis Poddubnyy

**Affiliations:** 1 Department of Gastroenterology, Infectious Diseases, and Rheumatology, Charité - Universitätsmedizin Berlin, Hindenburgdamm 30, 12203 Berlin, Germany; 2 Parc Taulí Hospital Universitari, I3PT, Universitat Autònoma de Barcelona, Sabadell, Barcelona Spain; 3 Epidemiology Unit, German Rheumatism Research Centre, Berlin, Germany; 4 Klinikum Bielefeld Rosenhöhe, Bielefeld, Germany

**Keywords:** Axial spondyloarthritis, mSASSS, Radiographic spinal progression, Radiographs, X-rays

## Abstract

**Background:**

To evaluate the performance of the extended modified Stoke Ankylosing Spondylitis Spine Score (mSASSS) incorporating information from anteroposterior (AP) lumbar radiographs as compared to the conventional mSASSS in detection of radiographic spinal progression in patients with axial spondyloarthritis (axSpA)

**Methods:**

A total of 210 patients with axSpA, 115 with radiographic axSpA (r-axSpA), and 95 with non-radiographic axSpA (nr-axSpA), from the GErman SPondyloarthritis Inception Cohort (GESPIC), were included in the analysis based on the availability of spinal radiographs (cervical spine lateral, lumbar spine lateral, and AP views), at baseline and year 2. Two trained readers independently scored lateral cervical and lumbar spine images according to the mSASSS system (0–3 per vertebral corner, 0–72 in total). In addition, all vertebral corners of vertebral bodies visible on lumbar AP radiographs (lower T12 to upper S1) were assessed according to the same scoring system that resulted in a total range for the extended mSASSS from 0 to 144. Reliability and sensitivity to detect radiographic spinal progression of the extended mSASSS as compared to the conventional mSASSS were evaluated.

**Results:**

The reliability of conventional and extended scores was excellent with intraclass correlation coefficients (ICCs) of 0.926 and 0.927 at baseline and 0.920 and 0.933 at year 2, respectively. The mean ± SD score for mSASSS and extended mSASSS at baseline were 4.25 ± 8.32 and 8.59 ± 17.96, respectively. The change score between baseline and year 2 was 0.73 ± 2.34 and 1.19 ± 3.73 for mSASSS and extended mSASSS, respectively. With the extended mSASSS, new syndesmophytes after 2 years were detected in 4 additional patients, new syndesmophytes or growth of existing syndesmophytes in 5 additional patients, and progression by ≥ 2 points in the total score in 14 additional patients meaning a 25%, 28%, and 46% increase in the proportion of patients with progression according to the respective definition as compared to the conventional score.

**Conclusions:**

Incorporation of lumbar AP radiographs in the assessment of structural damage in the spine resulted into detection of additional patients with radiographic spinal progression not captured by the conventional mSASSS score.

## Introduction

Axial spondyloarthritis (axSpA) can be classified into radiographic SpA (r-axSpA, also known as ankylosing spondylitis—AS) or non-radiographic SpA (nr-axSpA) depending on the presence or absence of definite radiographic sacroiliitis according to the grading system of the modified New York (mNY) criteria [1]. New bone formation with development of syndesmophytes between vertebrae represents the morphological substrate of structural damage in the spine in axSpA. Since development of structural damage is usually assessed on conventional radiographs, it is referred to as radiographic spinal progression. Structural damage in the spine and disease activity are two major determinants of spinal mobility and physical function in axSpA [2–4]. Currently, the modified Stoke Ankylosing Spondylitis Spine Score (mSASSS) [5] is considered as the gold standard for assessment of structural damage in the spine in axSpA [6]. Compared to the Bath Ankylosing Spondylitis Radiology Index (BASRI) [7] and SASSS [8], mSASSS has a better reliability and sensitivity to change [9].

The mSASSS assesses the presence of erosions, sclerosis, squaring, non-bridging syndesmophytes, and bony bridges affecting the anterior vertebral corners of the cervical and lumbar spine on radiographs in lateral views [5]. Previous studies suggested that anteroposterior (AP) view of the lumbar spine, which are frequently performed routinely, could provide additional information on the presence of structural damage in the spine [7, 9]. The added value of the AP lumbar radiographs in the assessment of radiographic spinal progression in axSpA as compared to lateral views only is, however, not known.

The aim of the current study was to evaluate the performance of the extended mSASSS score incorporating information of the AP lumbar radiographs compared to the conventional mSASSS.

## Patients and methods

### Patients

Patients with axSpA from the GErman SPondyloarthritis Inception Cohort (GESPIC) were included in the current analysis based on the availability of clinical data and radiographs at baseline and year 2. A detailed description of the entire cohort as well as of the radiographic subset has been reported previously [10–12]. Briefly, patients included in GESPIC had a definite clinical diagnosis of axSpA according to the treating rheumatologist with symptom duration of up to 5 years for the non-radiographic form and up to 10 years for the radiographic form of axSpA. The final classification as nr-axSpA or r-axSpA was performed based on the central reading of the radiographs as previously reported [10]. Treatment of the patients was defined by local rheumatologists and followed national and international guidelines; however, since the cohort had started prior to introduction of tumor necrosis factor inhibitors (TNFi) in the clinical practice, the vast majority of the patients were off TNFi therapy at inclusion and during first 2 years of observation.

### Radiographic assessments

Radiographs of cervical (lateral view) and lumbar spine (lateral and AP views) were obtained at baseline and after 2 years. Images were centrally collected, digitized, anonymized, and scored independently by two trained readers (DP and HH), who were blinded to the chronological order of the images and to all clinical data. According to the mSASSS, anterior corners of the vertebral bodies from lower C2 to upper T1 (cervical spine) and from lower T12 to upper S1 (lumbar spine) were scored as follows: 0 = normal, 1 = erosion, sclerosis, and/or squaring, 2 = non-bridging syndesmophyte, and 3 = bridging syndesmophyte, giving a range for the entire score from 0 to 72 [5]. No adjudication was performed. The final mSASSS score included in the analysis was calculated as a mean of the mSASSS scores of both readers. In addition, left and right, upper and lower vertebral corners of vertebral bodies visible on lumbar AP radiographs from lower Th12 to upper S1 were scored according the same grading system. The combination of the scores from both lateral cervical and lumbar views and lumbar AP views composed the “extended mSASSS” with a total range from 0 to 144. The final extended mSASSS was calculated as a mean of the scores of both readers. Syndesmophytes were considered to be present if both readers gave a score of ≥ 2 to a vertebral corner.

Missing scores of single vertebral corners were substituted with scores for the respective vertebral corners at other time point, if available, or with a score of zero if scores for both time points were missing.

### Data analysis

To assess the applicability of the extended mSASSS as a tool for the measurement of radiographic spinal progression, it was compared to the conventional mSASSS according to the three aspects of the Outcome Measures in Rheumatology (OMERACT) filter: feasibility, discrimination, and truth [13].

#### Feasibility

The feasibility aspect of the OMERACT filter refers to the pragmatic reality on applying or using a specific measure; which is decisive on its success: “Can the measure be applied easily, given constraints of time, money, and interpretability?” [13]. The feasibility was assessed in the context of time, radiation exposure, and cost excess associated with extended mSASSS compared to the conventional one.

#### Discrimination

The discrimination aspect addresses the question: “Does the measure discriminate between situations of interest?” This aspect of the OMERACT filter focuses on reliability and sensitivity to change [13].

We used the intraclass correlation coefficient (ICC) and Cohen’s kappa coefficient to measure the inter-observer reliability for continuous and categorical variables, respectively. The analysis was performed for the overall scores as well as separately for the components of the scores (cervical spine lateral view, lumbar spine lateral view, lumbar spine AP view). Additionally, a Bland and Altman plot was drawn and the smallest detectable change (SDC) was determined to assess the sensitivity to change of both methods (mSASSS and extended mSASSS). The SDC expresses the smallest change in scores that can be detected without measurement error, and it was calculated as follows:

SDC = 1.96 × SEM,

where SEM denotes the standard error of measurement of the change score obtained from a two-way analysis of variance by taking the square root of the error variance [14].

To measure the sensitivity to change, we used the following definitions of progression: (1) change of the absolute score (calculated as means of the scores of both readers), (2) change of the score by ≥ 2 points after 2 years, (3) development of at least one new syndesmophyte (score of 0 or 1 at baseline and score of 2 or 3 after 2 years at the same vertebral corner in the opinion of both readers), and (4) development of new syndesmophytes (as described above) or growth of existing syndesmophytes (score of 2 at baseline and 3 after 2 years at the same vertebral corner) in the opinion of both readers. The sensitivity to change was evaluated in the entire group and in r-axSpA and nr-axSpA subgroups. We also analyzed both status and progression scores in the components of the mSASSS and the extended mSASSS (cervical spine lateral view, lumbar spine lateral view, lumbar spine AP view). A variance component analysis was performed.

#### Truth

The truth aspect addresses the question: “Is the measure truthful, does it measure what is intended? Is the result unbiased and relevant?” [13]. Earlier studies showed an association between the mSASSS and spinal mobility/functional status in axSpA [2–4]. With a linear regression analysis, we investigated the relationship between both scores and spinal mobility (assessed by the Bath Ankylosing Spondylitis Metrology Index—BASMI, an original two-step definition) and function (assessed by the Bath Ankylosing Spondylitis Functional Index—BASFI).

Statistical analysis was performed with IBM SPSS Statistics version 24 (IBM, Armonk, NY, USA) and SAS version 9.4 (SAS Institute Inc., Cary, NC, USA).

## Results

A total of 210 patients with axSpA, 115 with r-axSpA and 95 with nr-axSpA, were included in the analysis based on availability of the full sets of radiographs: lumbar (AP and lateral views) and cervical (lateral view) spine at baseline and after 2 years. Demographic, clinical, and radiographic data of the patients at baseline and year 2 are presented in Table 1.

**Table 1:** Baseline demographic and clinical characteristics of the included patients with axial spondyloarthritis

Parameter	Non-radiographic axial SpA (*n* = 95)	Radiographic axial SpA (*n* = 115)	All patients (*n* = 210)
Age, mean ± SD years	38.7 ± 9.9	36.1 ± 11.0	37.3 ± 10.6
Symptom duration, mean ± SD years	3.1 ± 2.2	5.0 ± 2.8	4.2 ± 2.7
Duration since diagnosis, mean ± SD years	1.0 ± 1.3	2.0 ± 2.0	1.5 ± 1.8
Male sex, *n* (%)	32 (33.7)	75 (65.2)	107 (51.0)
HLA-B27 positive, *n* (%)	69 (73.4)	97 (84.3)	166 (79.4)
Peripheral arthritis, *n* (%)	16 (16.8)	15 (13.0)	31 (14.8)
Enthesitis^†^, *n* (%)	23 (24.2)	23 (20.0)	46 (21.9)
Uveitis, ever, *n* (%)	15 (15.8)	27 (23.5)	42 (20.0)
Psoriasis, ever, *n* (%)	11 (11.6)	17 (14.8)	28 (13.3)
Inflammatory bowel disease, ever, *n* (%)	1 (1.1)	3 (2.6)	4 (1.9)
Family history for SpA, *n* (%)	16 (16.8)	19 (16.5)	35 (16.7)
BASDAI, mean ± SD, 0–10	4.2 ± 2.0	3.8 ± 2.2	4.0 ± 2.1
BASFI, mean ± SD, 0–10	2.8 ± 2.2	3.0 ± 2.4	2.9 ± 2.3
Treatment with NSAIDs, *n* (%)	65 (68.4)	78 (67.8)	143 (68.1)
Treatment with csDMARDs, *n* (%)	26 (28.6)	35 (32.4)	61 (30.7)
Treatment with systemic steroids, *n* (%)	6 (6.7)	6 (5.6)	12 (6.1)
Treatment with a TNFα blocker, *n* (%)	1 (1.1)	4 (3.7)	5 (2.5)
Smoking, current, *n* (%)	24 (25.3)	39 (33.9)	63 (30.0)

† Twelve enthesitis sites of the lower limbs plus optional symptomatic sites elsewhere were assessed

*BASDAI* Bath Ankylosing Spondylitis Disease Activity Index, *BASFI* Bath Ankylosing Spondylitis Functional Index, *csDMARDs* conventional synthetic disease-modifying antirheumatic drugs, *NSAIDs* nonsteroidal antiinflammatory drugs, *SD* standard deviation, *SpA* spondyloarthritis, *TNFα* tumor necrosis factor α

### Feasibility

In this study, lumbar AP radiographs were available in all 210 patients that was a requirement of the inclusion in the radiographic subset. Although AP lumbar radiograph is a routine projection in the clinical practice, it increases the costs of the radiographic investigation of the spine by approximately 50% and the time required for the reading according to the extended mSASSS by 50–100% (since additional 24 vertebral corners should be assessed) as compared to conventional mSASSS. Furthermore, AP lumbar radiographs are associated with additional radiation exposure, increasing the overall exposure associated with the assessment by approximately 60–70%.

### Discrimination

The status scores and progression data for the both scores is presented in Table 2. The mean ± standard deviation (SD) score at baseline for the whole group included in the analysis was 8.59 ± 17.96 and 4.25 ± 8.32 for the extended mSASSS and conventional mSASSS, respectively. The absolute change score between baseline and year 2 was 1.19 ± 3.73 and 0.73 ± 2.34 for the extended mSASSS and conventional mSASSS, respectively (Table 2). In the separate analysis of the components of the score, the progression rates were comparable across the components (Table 3).

**Table 2:** Status and change scores of mSASSS and extended mSASSS

	All patients (*n* = 210)		Radiographic axial SpA (*n* = 115)		Non-radiographic axial SpA (*n* = 95)	
	mSASSS	Extended mSASSS	mSASSS	Extended mSASSS	mSASSS	Extended mSASSS
Status score at baseline, mean ± SD	4.2 ± 8.3	8.6 ± 18.0	5.9 ± 10.3	12.8 ± 22.5	2.3 ± 4.2	3.5 ± 7.4
Status score at year 2, mean ± SD	5.0 ± 9.6	9.8 ± 20.0	6.8 ± 11.7	14.5 ± 24.9	2.8 ± 5.3	4.1 ± 8.9
Change score, mean ± SD	0.7 ± 2.3	1.2 ± 3.7	1.0 ± 2.8	1.7 ± 4.6	0.5 ± 1.6	0.6 ± 2.2
Smallest detectable change	4.1	6.4	4.7	7.5	3.3	4.6
Change score ≥ smallest detectable change, *n* (%)	14 (6.7)	12 (5.7)	7 (6.1)	8 (7.0)	4 (4.2)	6 (6.3)
Presence of syndesmophytes at baseline, *n* (%)	48 (22.9)	64 (30.5)	35 (30.4)	48 (41.7)	13 (13.7)	16 (16.8)
Presence of syndesmophytes at year 2, *n* (%)	50 (23.8)	65 (31.0)	36 (31.3)	50 (43.5)	14 (14.7)	15 (15.8)
Total number of syndesmophytes at baseline, mean ± SD	0.9 ± 2.6	1.9 ± 5.2	1.5 ± 3.3	3.2 ± 6.6	0.3 ± 1.1	0.5 ± 1.9
Total number of syndesmophytes at year 2, mean ± SD	1.1 ± 3.1	2.2 ± 6.0	1.7 ± 3.8	3.5 ± 7.6	0.4 ± 1.4	0.6 ± 2.4
Change score ≥ 2 points after 2 years, *n* (%)	30 (14.3)	44 (21.0)	23 (20.0)	35 (30.4)	7 (7.4)	9 (9.5)
Development of new syndesmophytes after 2 years, *n* (%)	16 (7.6)	20 (9.5)	13 (11.3)	17 (14.8)	3 (3.2)	3 (3.2)
Development of new /growth of existing syndesmophytes after 2 years, *n* (%)	18 (8.6)	23 (11.0)	15 (13.0)	20 (17.4)	3 (3.2)	3 (3.2)

*mSASSS* modified Stoke Ankylosing Spondylitis Spine Score, *SD* standard deviation

**Table 3:** Status and change scores of the components of the mSASSS and the extended mSASSS

	Cervical spine lateral view	Lumbar spine lateral view	Lumbar spine AP view
Change score ≥ 2 points after 2 years, *n* (%)	29 (13.8)	18 (8.6)	25 (11.9)
Total number of syndesmophytes at baseline, mean ± SD	0.5 ± 1.6	0.4 ± 1.5	1.0 ± 3.0
Total number of syndesmophytes at year 2, mean ± SD	0.6 ± 1.7	0.6 ± 1.8	1.1 ± 3.3
Development of new syndesmophytes after 2 years, *n* (%)	8 (3.8)	8 (3.8)	9 (4.4)
Development of new/growth of existing syndesmophytes after 2 years, *n* (%)	8 (3.8)	10 (4.8)	10 (4.8)

mSASSS includes scores of the cervical and lumbar lateral views; extended mSASSS includes additionally the lumbar spine AP view

*AP* anteroposterior, *mSASSS* modified Stoke Ankylosing Spondylitis Spine Score, *SD* standard deviation

There was an excellent agreement between readers on the status scores with ICCs of 0.93 (95% CI 0.91–0.94) and 0.93 (95% CI 0.90–0.94) at baseline and 0.93 (95% CI 0.91–0.95) and 0.92 (95% CI 0.90–0.94) at year 2 for the extended and conventional mSASSS, respectively. The agreement for the change score was moderate with ICC of 0.45 (95% CI 0.33, 0.55) and 0.43 (95% CI 0.31, 0.53) for the extended and conventional mSASSS, respectively. For the outcome progression by ≥ 2 points after 2 years, there was a fair agreement for both methods: Cohen’s kappa 0.20 (95% CI 0.06, 0.34) and 0.21 (95% CI 0.06, 0.36) for extended and conventional mSASSS, respectively. The agreement for development of new syndesmophytes was moderate with Cohen’s kappa of 0.39 (95% CI 0.28, 0.57) and of 0.43 (95% CI 0.28, 0.57) for extended and conventional mSASSS, respectively. The agreement was moderate also for the outcome development of new syndesmophytes or growth of existing syndesmophytes with Cohen’s kappa of 0.44 (95% CI 0.29, 0.58) and 0.42 (95% CI 0.29, 0.55) for the extended and conventional mSASSS, respectively. An analysis of the score components revealed a comparable variability of the scores obtained in the cervical spine and in both planes of the lumbar spine (Table 4). The variance component analysis revealed that the patient-related variance contributed most to the total variance of both mSASSS and extended mSASSS score (Table 5); the same finding was observed when the variance of score components was analyzed separately (Table 6).

**Table 4:** Inter-observer reliability of the components of the mSASSS and the extended mSASSS

	Cervical spine lateral view	Lumbar spine lateral view	Lumbar spine AP view
ICC for the status score at baseline	0.91 (0.88, 0.93)	0.91 (0.88, 0.93)	0.86 (0.83, 0.90)
ICC for the status score at year 2	0.86 (0.82, 0.89)	0.92 (0.90, 0.94)	0.88 (0.84, 0.90)
ICC for the change score	0.49 (0.39, 0.59)	0.43 (0.32, 0.54)	0.30 (0.19, 0.44)
Kappa for the change score ≥ 2 points after 2 years	0.13 (− 0.04, 0.29)	0.38 (0.18, 0.57)	0.36 (0.18, 0.54)
Kappa for the development of new syndesmophytes after 2 years	0.38 (0.20, 0.55)	0.59 (0.42, 0.77)	0.40 (0.24, 0.57)
Kappa for the development of new/growth of existing syndesmophytes after 2 years	0.34 (0.17, 0.52)	0.62 (0.46, 0.79)	0.48 (0.32, 0.64)

Data are presented as intraclass correlation coefficient (ICC) and Cohen’s kappa coefficient to measure the inter-observer reliability for continuous and categorical variables, respectively, with a 95% CI

*AP* anteroposterior, *mSASSS* modified Stoke Ankylosing Spondylitis Spine Score, *CI* confidence interval, *ICC* intraclass correlation coefficient

**Table 5:** Inter-observer reliability of the mSASSS and the extended mSASSS, expressed in variance components

	Status score at baseline			Status score at year 2			Change score		
	Residual variance component	Observer variance component	Patient variance component	Residual variance component	Observer variance component	Patient variance component	Residual variance component	Observer variance component	Patient variance component
mSASSS	7.45	0.03	92.52	7.95	0.05	92.00	57.42	0.00	42.58
Extended mSASSS	7.28	0.01	92.71	6.67	0.00	93.33	54.99	0.00	45.01

*mSASSS* modified Stoke Ankylosing Spondylitis Spine Score

**Table 6:** Inter-observer reliability of the components of the mSASSS and the extended mSASSS, expressed in variance components

	Cervical spine lateral view			Lumbar spine lateral view			Lumbar spine AP view		
	Residual variance component	Observer variance component	Patient variance component	Residual variance component	Observer variance component	Patient variance component	Residual variance component	Observer variance component	Patient variance component
Status score at baseline	8.87	0.06	91.07	9.05	0.00	90.95	13.23	0.22	86.55
Status score at year 2	13.71	0.19	86.10	7.49	0.00	92.51	12.14	0.13	87.73
Change score	50.49	0.18	49.33	57.11	0.00	42.89	69.54	0.00	30.46

*AP* anteroposterior, *mSASSS* modified Stoke Ankylosing Spondylitis Spine Score

**Fig. 1:**
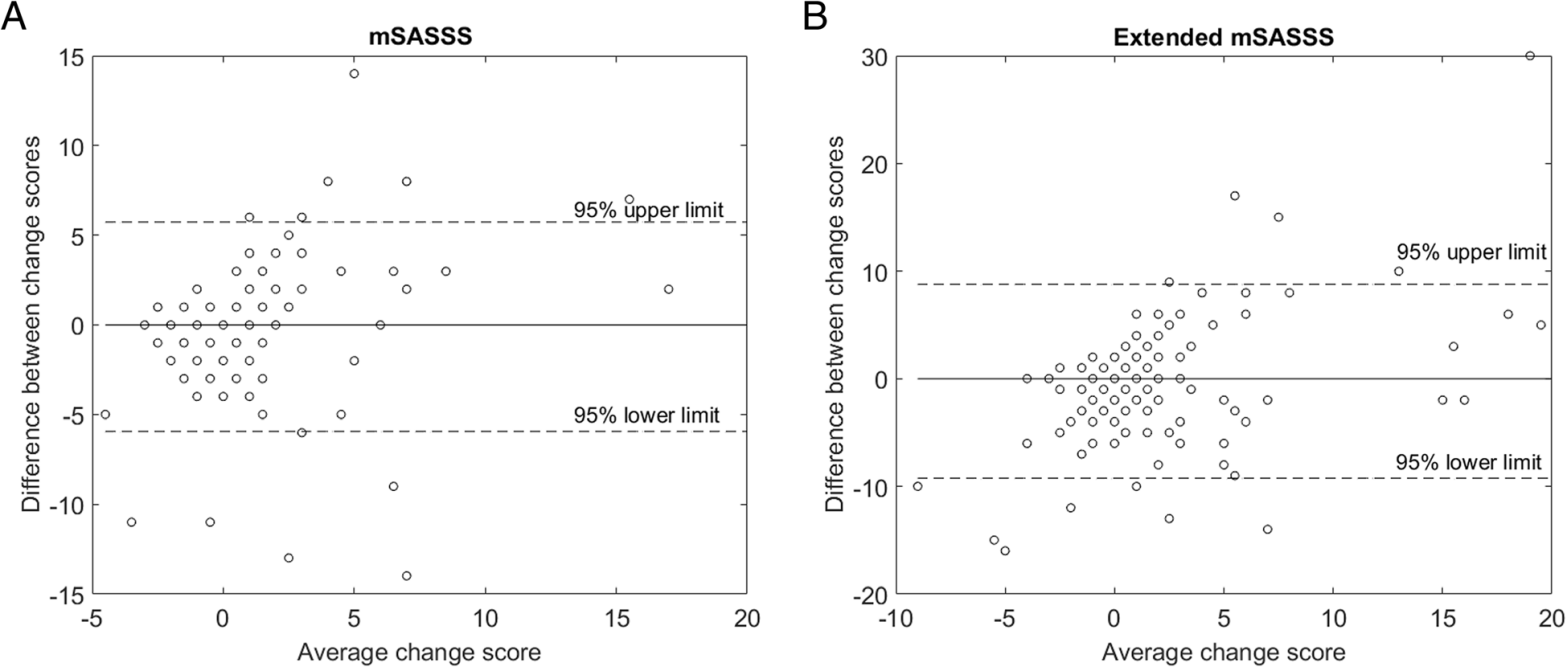
Bland and Altman plots for mSASSS (**a**) and extended mSASSS (**b**)

The Bland and Altman plots illustrating the inter-reader reliability of the extended and conventional scores are presented in Fig. 1. The SDC was higher for the extended mSASSS than for the conventional one (Table 2). Regarding the presence of syndesmophytes in the opinion of both readers at baseline, extended mSASSS detected syndesmophytes in 64 patients (30.5%) and conventional mSASSS in 48 patients (22.9%); the mean total number of syndesmophytes at baseline was 1.92 ± 5.24 and 0.94 ± 2.61 for the extended mSASSS and conventional mSASSS, respectively. Importantly, the extended mSASSS detected new syndesmophytes in 4 additional patients, new syndesmophytes or progression of existing syndesmophytes in 5 additional patients, and progression by ≥ 2 units in the total score in 14 additional patients meaning a 25%, 28%, and 46% increase in the proportion of patients with progression according to the respective definition as compared to the conventional mSASSS (Table 2).

When the two subgroups of patients (r-axSpA and nr-axSpA) were analyzed separately, the mean ± SD score at baseline as well as progression rates over 2 years were lower in the nr-axSpA group for both scoring systems (Table 2).

### Truth

In the univariable analysis, both extended and conventional mSASSS were significantly associated with BASMI and BASFI (Table 7). In the multivariable analysis that was adjusted for disease activity parameters (BASDAI and CRP), the association of both scores with spinal mobility/function remained significant (Table 7).

**Table 7:** Association between mSASSS or extended mSASSS score with BASFI or BASMI at baseline in the linear regression analysis

	Univariable analysis	Multivariable model 1*	Multivariable model 2*
	*β* (95% CI)	*β* (95% CI)	*β* (95% CI)
Outcome: BASFI			
mSASSS	0.046 (0.008, 0.083)	0.033 (0.010, 0.056)	–
Extended mSASSS	0.021 (0.003, 0.038)	–	0.014 (0.004, 0.025)
Outcome: BASMI			
mSASSS	0.093 (0.068, 0.117)	0.082 (0.059, 0.106)	–
Extended mSASSS	0.045 (0.034, 0.056)	–	0.040 (0.029, 0.050)

*Adjusted for BASDAI and CRP at baseline

*BASDAI* Bath Ankylosing Spondylitis Disease Activity Index, *BASFI* the Bath Ankylosing Spondylitis Functional Index, *BASMI* Bath Ankylosing Spondylitis Metrology Index, *mSASSS* modified Stoke Ankylosing Spondylitis Spine Score

## Discussion

In the present study, we evaluated the added value of incorporation of AP lumbar radiographs in the mSASSS scoring system for the assessment of structural damage in the spine in patients with axSpA. The extended mSASSS showed a good reliability and provided some improvement of the detection of radiographic spinal progression in patients with axSpA as compared to the conventional mSASSS that has to be weighed against additional costs, additional radiation exposure (although AP lumbar radiographs are often performed routinely), and additional time investment associated with reading of additional 24 vertebral corners.

Extended mSASSS can be considered as supplementary to the conventional mSASSS as it adds information from the AP lumbar spine view. The information obtained from the AP lumbar radiograph can also be to some extent redundant, since the same structural damage (same syndesmophyte) can be visible on both lateral and AP views. This fact explains, for instance, a larger difference in status scores (the extended mSASSS was double as high as the conventional one) than in percentages of patients with syndesmophytes (30.5% with extended mSASSS vs. 22.9% with conventional mSASSS at baseline).

The most relevant question is, however, if the extended mSASSS is able to detect patients with radiographic spinal progression not captured by the conventional method. Indeed, with the extended mSASSS, new syndesmophytes after 2 years were detected in 4 additional patients, new syndesmophytes or growth of existing syndesmophytes in 5 additional patients, and progression by ≥ 2 points in the total score in 14 additional patients that gave a 25%, 28%, and 46% increase in the proportion of patients with progression according to the respective definition as compared to the conventional score. These proportions were even higher in patients with r-axSpA meaning that in more advanced disease the added value of the lumbar AP assessment might be greater than in early disease [15]. In our analysis, the subgroup of patients with r-axSpA had a higher level of structural damage at baseline and higher progression rates after 2 years compared to nr-axSpA patients. At the same time, in advanced disease with extended ankylosis, one can expect a larger extent of structural damage that would be captured on both lateral and AP views that might result into a reduction of the added value of the AP lumbar radiographs.

The extended mSASSS—similarly to the conventional mSASSS—captures only a part of the structural damage visible on radiographs. Syndesmophytes with posterior localization or in the lateral aspects of the cervical spine, as well as involvement of posterior structures (i.e., facet joints), are not captured by any score. In the majority of cases, however, structural damage develops more or less simultaneously in different aspects of the spinal column that allows the assumption that the mSASSS/extended mSASSS is as a proxy for the overall structural damage in the spine. In the univariable and multivariable regression analyses, both scores showed a significant association with spinal mobility (BASMI) and function (BASFI) that confirms the construct validity of both scores as parameters truly reflecting structural damage in the spine in axSpA. A potentially important limitation of any radiographs is the exclusion of the thoracic spine from scoring due to anatomical overlap with other thoracic structures. However, active inflammatory [16] and structural changes [17] seem to occur here at least as frequent as in other spine regions. For this reason, another modification of the mSASSS was proposed by Baraliakos et al: the Radiographic AS Spinal Score (RASSS), which adds to the mSASSS the score of the lower thoracic vertebrae (T10–T12), under the hypothesis that most of progression is found in these segments, and the quantification of new bone formation is superior to the conventional mSASSS [18]. Nevertheless, a recent study that compared the mSASSS with the RASSS [19] concluded the limitation of the RASSS in the feasibility aspect (in 64% of radiographs of the lumbar spine, the lower thoracic vertebrae were not visible, and therefore, the RASSS could not be calculated). Due to low reliability, no thoracic spine radiographs and no AP cervical radiographs were collected in GESPIC for the assessment of structural damage.

Other imaging methods without technical limitations of plain radiographs—such as low-dose computed tomography (CT)—have a potential to become the primary methods of radiographic spinal progression in axSpA in the future [17].

## Conclusions

The incorporation of the AP radiographs of the lumbar spine in the assessment of structural damage in the spine provides improvement of detection of radiographic spinal progression in axSpA.

## Abbreviations

AP: Anteroposterior
AS: Ankylosing spondylitis
axSpA: Axial spondyloarthritis
BASDAI: Bath Ankylosing Spondylitis Disease Activity Index
BASFI: Bath Ankylosing Spondylitis Functional Index
BASMI: Bath Ankylosing Spondylitis Metrology Index
BASRI: Bath Ankylosing Spondylitis Radiology Index
CI: Confidence interval
CRP: C-reactive protein
CT: Computed tomography
GESPIC: GErman SPondyloarthritis Inception Cohort
ICCs: Intraclass correlation coefficients
mNY: Modified New York
mSASSS: Modified Stoke Ankylosing Spondylitis Spine Score
nr-axSpA: Non-radiographic axial spondyloarthritis
OMERACT: Outcome Measures in Rheumatology
r-axSpA: Radiographic axial spondyloarthritis
SD: Standard deviation
SDC: Smallest detectable change
SEM: Standard error of measurement
TNFi: Tumor necrosis factor inhibitor
